# Antecedent life events of binge-eating disorder

**DOI:** 10.1016/j.psychres.2005.10.006

**Published:** 2006-05-30

**Authors:** Kathleen M. Pike, Denise Wilfley, Anja Hilbert, Christopher G. Fairburn, Faith-Anne Dohm, Ruth H. Striegel-Moore

**Affiliations:** a Department of Psychiatry, Unit 98, Columbia University, 1051 Riverside Drive, NY, 10032, USA; b Department of Psychology, Washington University in St. Louis, St. Louis, MO, USA; c Department of Psychiatry, Oxford University, Oxford, UK; d Graduate School of Education and Allied Professions, Fairfield University, Fairfield, CT, USA; e Department of Psychology, Wesleyan University, Middletown, CT, USA

**Keywords:** Eating disorders, Binge-eating disorder, Binge-eaters, Risk factors, Etiology, Life events, Life stress

## Abstract

The present study investigated the occurrence of life events preceding the onset of disturbed eating in binge-eating disorder (BED). In a case-control design, 162 matched pairs of black and white women with BED and women with no current psychiatric disorder, and 107 matched pairs of women with BED and a current general psychiatric disorder were recruited from the community for the New England Women's Health Project. Life events in the year before the onset of disturbed eating were assessed retrospectively with an investigator-based interview. Women with BED reported exposure to a significantly greater number of life events during the year before onset of eating disturbances than both the non-psychiatric and psychiatric control women during the same period of time in their lives. Women with BED had a significantly higher risk of exposure to certain specific life events (e.g., critical comments about shape, weight, or eating; stress related to work, school or other sources; major changes in life circumstances and relationships; physical abuse; and feeling unsafe in a variety of settings) than the non-psychiatric control women, while differences between the BED and the psychiatric control group were less marked. There was no evidence for race-specific exposure to antecedent life events. The results suggest that a greater number and certain specific types of life events increase risk for the subsequent development of BED.

## 1 Introduction

The inclusion of binge-eating disorder (BED) in the Diagnostic and Statistical Manual of Mental Disorders (DSM-IV; American Psychiatric Association, 1994) catalyzed research on the etiology of this disorder. Two comprehensive studies of psychosocial risk factors converge to document a multi-determined etiology of BED (Fairburn et al., 1998; Striegel-Moore et al., 2005). Specifically, those community-based, case-control investigations used a retrospective interview to assess a range of adverse childhood experiences, disturbances in family functioning and psychopathology, and disturbances in individual and family eating and weight patients that increase risk for the subsequent development of BED. Findings from the New England Women's Health Project (NEWHP; Striegel-Moore et al., 2005) further suggest that risk factors are comparable for black and white women.

Although the extant data indicate that vulnerability for the development of BED is multidetermined, why an individual develops BED at a certain moment in time was not addressed in these reports. Yet, the role of antecedent life events in the onset of psychopathology is well established, particularly for depression and anxiety disorders (Bebbington et al., 1993; Kendler et al., 1999, 2003). Within the field of eating disorders, a number of studies suggest that stressful life events often closely antedate the onset of anorexia nervosa (AN) and bulimia nervosa (BN; for an overview, see Jacobi et al., 2004). Stressful life events associated with the onset of adolescent AN include significant family conflict and disruption, change of school or home, and increased academic pressure (Margo, 1985; Horesh et al., 1995). Compared with early-onset AN, late-onset AN may be antedated by a greater number of stressful life events, of which family conflict or loss and medical illness are the most notable (Mynors-Wallis et al., 1992). The onset of BN has been associated with losses and separations from significant others (Pyle et al., 1981; Lacey et al., 1986), interpersonal problems with family and friends, health problems (Schmidt et al., 1993, 1997), and threat to physical safety (Welch et al., 1997). Moreover, some data suggest that severe life-event stresses may have particular potency in increasing risk for both AN and BN (Schmidt et al., 1997).

To our knowledge, the occurrence of life events before the onset of BED has not been studied. Thus, the two primary questions addressed in this investigation are the following: 1) Do individuals with BED experience a significantly greater number of stressful life events in the year immediately preceding the emergence of their eating problems compared with matched individuals with either other current general psychiatric disorders (PC group) or no current psychiatric disorder (NC group) at the same stage in their lives? 2) Are particular types of antecedent life events especially likely to precede the onset of BED? In addition, secondary analyses were conducted to assess the potential moderating role of race, weight status, comorbid psychiatric disorder, and age.

## 2 Methods

The data reported in this study derive from an interview-based assessment conducted under the auspices of the NEWHP that compared the life experience of black and white women with BED before the onset of significant eating disturbance with those of PC and NC control groups with respect to the same period of time in their lives. A detailed description of the overall methods of the NEWHP is provided in Pike et al. (2001) and Striegel-Moore et al. (2005). Methodological details pertinent to this report are included below.

### 2.1 Recruitment

Two strategies of recruitment were used 1) a consumer data base was used to contact approximately 10,000 potential participants and 2) an advertising campaign invited women to participate in a study of women's health. There was no selective ethnic bias in the results of the recruitment strategies (51.8% of the white women and 52.4% of the black women were recruited through the consumer database). The advertising campaign yielded 76.7% of the BED cases whereas the consumer data base yielded 80.7% of the healthy controls. The consumer data base and the advertising campaign yielded approximately equal percentages of PC cases (53.7% and 46.3% respectively).

Fifteen-minute screening interviews assessed study eligibility (participation rate: 91%). Exclusion criteria were age over 40 or under 18 years, physical conditions known to influence eating habits or weight, current pregnancy, presence of psychotic disorder, not being black or white, or not being born in the United States. On the basis of US census criteria, information about race/ethnicity was obtained at the end of the call and eligible women were invited to complete the diagnostic (First et al., 1995) and risk factor assessment interviews (Fairburn et al., 1998). Overall participation rates for black and white women in the NEWHP, respectively, were as follows: BED 84.8%, 85.2%; NC 62.7%, 74.5%; PC 76.6%, 73.9%.

The institutional review boards at Wesleyan and Columbia Universities reviewed and approved this study. Informed consent was secured verbally for the telephone screening interview, and written informed consent was secured in person for all individuals who ultimately participated in the study. Study participation and data collection were confidential, and participants received payment for their time.

### 2.2 Participants

The sample included 162 women whose primary psychiatric diagnosis at the time of assessment was (current) BED. Mean duration of BED was 9.49 ± 8.01 years (range: 0–30 years). As expected, BED diagnosis was associated with psychiatric comorbidity (lifetime), especially mood disorders (mood disorders: *n* = 111 [68.5%], anxiety disorder: *n* = 55 [34.0%], substance disorder: *n* = 64 [39.5%], any Axis I disorder: *n* = 137 [84.6%]) and, in a minority of cases, a history of BN (*N* = 19; 11.7%) and AN (*n* = 1; 0.6%). The BED group was matched for race, age, and education to the NC group of 162 women and the PC group of 107 women (see Table 1).

The PC group included individuals with a current DSM-IV Axis I psychiatric disorder (mood disorder: *n* = 53 [49.5%], anxiety disorder: *n* = 47 [43.9%], substance disorder: *n* = 4 [3.7%], other Axis I disorders: *n* = 3 [2.9%]), but without a history of clinically significant eating disorder symptoms. The NC group did not meet criteria for any current Axis I psychiatric disorder and had no history of binge-eating or extreme weight-control behavior. History of DSM-IV Axis I psychiatric disorders included mood disorders (*n* = 8, 4.9%), anxiety disorders (*n* = 15, 9.3%), and substance disorder (*n* = 11, 6.8%). Although matched on age (± 2 years), the BED group was approximately 9 months older than the NC group and 13 months older than the PC group, both differences being statistically significant or marginally significant using an adjusted *α* of 1% (Table 1; *P* = 0.002 or 0.025). As would be expected, the BED group reported a significantly higher mean body mass index (BMI) than both control groups (Table 1; both *P*'s < 0.001). Numbers of participants with a psychiatric diagnosis before the index age were not significantly different in the BED vs. PC group (BED: *n* = 39 [37.1%], PC: *n* = 47 [43.9%]; McNemar *χ*_1_^2^ = 1.11, *P* = 0.291), but as expected, more BED participants than NC participants revealed a psychiatric diagnosis before the index age (BED: *n* = 64 [40.0%], NC: *n* = 11 [6.8%]; McNemar *χ*_1_^2^ = 40.64, *P* < 0.001).

### 2.3 Assessment

#### 2.3.1 Clinical diagnosis

We assessed current and lifetime psychiatric disorders with the Structured Clinical Interview for DSM-IV Axis I Disorders (SCID-IV; First et al., 1995). Eating disorder diagnoses and psychopathology were further assessed using an abbreviated diagnostic version of the Eating Disorder Examination (EDE; Fairburn and Cooper, 1993), an investigator-based interview.

#### 2.3.2 Determination of “index age”

At the outset of the EDE interview, the age at onset of clinically significant eating disturbance (i.e., regular binge eating, purging, or strict dieting) was determined in the BED cases based on a careful, behaviorally based history (Fairburn et al., 1997). This *index age* was subsequently used as a chronological marker. Subsequent assessment focused on the period of 12 months immediately preceding the participant's index age*,* ensuring that the life events assessed antedated the onset of clinically significant eating pathology. The identification of life events in the NC and PC groups focused on the same year of age as the matched BED case in order to assure assessment of the same developmental window across the three groups.

#### 2.3.3 Antecedent life events

A modified version of the Oxford Risk Factor Interview (RFI; Fairburn et al., 1998) assessed a range of antecedent life events. The RFI assessment of antecedent life events includes 17 items that use behavioral definitions of key concepts to minimize memory or reconstruction biases frequently associated with retrospective reporting (Brown and Harris, 1989; Brewin et al., 1993; Paykel, 1997). The structure of the interview is such that each event was enquired for unless it clearly did not apply (e.g., divorce for someone who never married). If a respondent endorsed an event, the specific timing and circumstances were queried to ascertain a level of certainty regarding the occurrence of the event.

Table 2 lists the life events assessed over the year immediately antedating the onset of disturbed eating. To reduce the number of variables, two composite variables were computed based on thematic content. “Major Stress from School, Work or Other Source” was constructed from two separate items: 1) major pressure or stress at school or work; 2) any other stress or pressure. “Concerns about Safety” was constructed from four items: 1) worried for personal safety; 2) feeling unsafe at home; 3) feeling unsafe in your neighborhood; 4) feeling unsafe at school or work. This resulted in a total of 13 items being entered into the statistical analyses. All the items were initially rated on a 4-point scale, but for the statistical analyses the ratings were dichotomized: 0 = no event occurred (initially coded 0, 1, 2, or 3) vs. 1 = definitely occurred (initially coded 4).

### 2.4 Data analysis

Chi-square analyses for matched samples were used to examine the proportion of single life events in the BED group vs. the NC group and in the BED group vs. the PC group (McNemar tests). An overall measure of exposure to life events was obtained by summing the number of events to which each subject had been exposed. Subjects were grouped into four groups representing their degree of exposure to significant life events (0, 1, 2, and 3 or more events). Group differences were analyzed using Stuart–Maxwell *χ*^2^ tests of overall marginal homogeneity. For post-hoc analyses, McNemar tests were performed on single proportions. Odds ratios (OR) were computed to estimate the relative risk of exposure to types and numbers of life events. The area under the curve statistic (AUC) was used as a measure of effect size indicating the probability that a BED case would be more likely to endorse an event than a control case (AUC% = 50(*P*_case_ − *P*_control_ + 1) with P indicating the proportion of cases or controls endorsing an antecedent; Kraemer et al., 2001). According to Cohen's classification of effect sizes, AUC% < 55.6% is very low; 55.6% ≤ AUC% < 63.8% is low; 63.8% ≤ AUC% < 71.4% is medium; and AUC% ≥ 71.4% is large (Cohen, 1988). Comparisons of black and white women within the BED, NC, and PC groups were performed using Pearson's *χ*^2^ tests. To determine whether the odds of exposure to antecedent life events were moderated by race or weight status (i.e., obesity, BMI ≥ 30.0 kg/m^2^), black vs. white women and obese vs. non-obese women were compared using Pearson's *χ*^2^ tests within the BED, NC, and PC groups. In addition, associations between age and exposure to number or types of antecedents were analyzed using point–biserial correlation coefficients, and the potential effect of a comorbid depressive mood disorder (lifetime) on the reporting of antecedent life stress in the BED group was analyzed using Pearson's *χ*^2^ tests. For all analyses, an adjusted two-tailed *α* of 1% was used (*P* < 0.01).

## 3 Results

### 3.1 BED vs. non-psychiatric control group: number of antecedents

As indicated in Table 3, the BED group reported a significantly greater number of life events than the NC group in the year preceding onset of their eating disturbance (Stuart Maxwell *χ*_3_^2^ = 64.19, *P* < 0.001). In comparison with the NC group, post-hoc analyses indicated that a higher percentage of the BED women reported three or more life events stressors during the year before their index age (*n* = 98 [60.5%] vs. *n* = 34 [21.0%]; McNemar tests, *P* < 0.001), while a lower percentage reported no life events (*N* = 10 [6.2%] vs. *n* = 65 [40.1%]; *P* < 0.001) or a single life event (*n* = 20 [12.3%] vs. *n* = 38 [23.5%]; *P* = 0.006). Exposure to a greater number of life events was associated with a six times higher risk of BED (3 or more events: OR = 5.8), while exposure to no or a lower number of events indicated a reduced risk (0 and 1 events: both ORs < 1.0). Exposure to three or more life events was an antecedent of medium potency (AUC% = 69.8%).

### 3.2 BED vs. non-psychiatric control group: types of antecedents

Table 2 reports the group comparisons of the types of events reported during the year before the index age. The women with BED reported significantly more life event stressors than the NC women during the year preceding the index age. Specifically, they reported a significantly greater number of major changes in life circumstances and relationships (i.e., major house move; death of a close relative, friend or partner; change in family structure; end of relationship with boyfriend/partner; all *P*'s < 0.005). Women with BED further reported significantly more critical comments about shape and weight than NC women and significantly greater risk of physical abuse (both *P*'s < 0.001). Weight- and shape-related criticism was seven times more likely for BED women than for NC women (OR = 7.4). Physical abuse was six times more likely to be reported by women with BED than by NC women (OR = 6.3). Women with BED also described significantly more work, school or other-related stress and more concerns about safety or feeling unsafe in a variety of settings than the NC group (both *P*'s < 0.001). Critical comments about shape and weight and work, school or other-related stress were antecedents of medium potency (AUC% = 67.1% or 66.4%, respectively).

### 3.3 BED vs. psychiatric control group: number of antecedents

In terms of exposure to the overall number of antecedents, the comparisons of the psychiatric and BED groups follow a similar pattern to that described for the BED vs. NC group (see Table 3). Again, the BED women reported exposure to an overall greater number of life events than the PC group (Stuart Maxwell *χ*_3_^2^ = 22.28, *P* < 0.001). A post-hoc McNemar test indicated that three or more life events were more frequently endorsed by the BED group than by the PC group (BED: *n* = 67 [62.6%]; NC: *n* = 33 [30.8%]; *P* < 0.001), while one life event was less frequently reported (10 [9.3%] vs. 26 [24.3%]; *P* = 0.004). Exposure to a greater number of life events was associated with a relatively higher risk of BED (3 or more events: OR = 3.9), while exposure to no or one event indicated a low risk (0 and 2 events: both OR's < 1.0).

### 3.4 BED vs. psychiatric control group: types of antecedents

Compared with the PC group, the BED group reported significantly more frequent exposure to several life events in the year preceding index age (see Table 2). Specifically, the BED group reported more frequent occurrence of change in family structure (*P* = 0.005) and of critical comments about shape and weight (*P* = 0.001). Also, compared with the PC group, the BED group showed a trend for more frequent ending of relationships with boyfriend/partner (*P* = 0.015) and concerns about safety or feeling unsafe in a variety of settings (*P* = 0.016). The two groups did not differ in their reported risk of exposure to physical or sexual abuse during the year preceding the index age.

### 3.5 Race, weight status, and age as moderators of antecedents

To determine whether risk of exposure to antecedent life events was a function of race, black vs. white women were compared within each diagnostic group (BED and NC—black: 60 [37.0%], white: 102 [63.0%]; BED and PC—black: 21 [19.6%], white: 86 [80.4%]). Black and white women did not differ in the overall number of reported stressful life events in the year preceding the index age (black vs. white: BED: *χ*_3_^2^ = 1.10, *P* = 0.777; NC: *χ*_3_^2^ = 0.95, *P* = 0.814; PC: *χ*_3_^2^ = 1.61, *P* = 0.656). Analyses comparing black and white women on each antecedent resulted in only one life event stressor that was endorsed at significantly different rates by black and white women. In the BED group, black women reported significantly higher risk of pregnancy during the year before the onset of eating disorder symptoms than white women (*χ*_1_^2^ = 10.82, *P* < 0.001) and the NC group showed a trend in the same direction (Fisher's exact test, *P* = 0.04). As these data suggest that pregnancy is specific for black vs. white race rather than for case status, results provided no evidence for antecedents of BED as a function of race in black vs. white women. Further, there were no significant interactions between group and race on index age, current age, or BMI (two-way repeated measures analyses of Group [BED vs. NC or BED vs. PC] × Race [black vs. white], *P* > 0.01 for all), and educational level did not differ between black and white women within each group (*χ*^2^ tests, all *P'*s > 0.01).

Further, to determine the potential effect of obese weight status on antecedents, comparisons of obese vs. non-obese women were performed within each diagnostic group (see Table 1). In all diagnostic groups, obese vs. non-obese women did not show any differences in overall number of life events (BED: *χ*_3_^2^ = 2.54, *P* = 0.469; NC: *χ*_3_^2^ = 9.61, *P* = 0.022; PC: *χ*_3_^2^ = 2.44, *P* = 0.487). Concerning specific types of life event stressors, critical comments about weight, shape, or eating were more frequently reported by obese women than by non-obese women in the BED group (BED: *χ*_1_^2^ = 7.81, *P* = 0.005), but no other differences between obese and non-obese women occurred in this group (*P*'s > 0.01). In the NC group, obese women reported higher odds of pregnancy (*χ*_1_^2^ = 7.54, *P* = 0.006) and of illness of a significant other (*χ*_1_^2^ = 8.28, *P* = 0.004). In the PC group, obesity did not moderate exposure to specific types of life event stressors (all *P*'s > 0.05). These results indicate that exposure to critical comments about weight, shape, and eating is moderated by obese weight status in the BED group. In addition, while the overall number of antecedent life events does not differ by weight status, for some specific types of life event stressors, i.e., pregnancy or illness of a close person, influence of obesity depends on case status.

Lastly, for determining a potential effect of age on exposure to antecedents, a correlational analysis between age and exposure to number or types of antecedents did not reveal any significant association in either study group (point–biserial correlation coefficients, all *P*'s > 0.01). (Detailed tables for moderator analyses are available upon request.)

### 3.6 Association between depression and antecedents in women with BED

Statistical analyses on the potential effect of a comorbid mood disorder on the reporting of exposure to antecedent life stress in the BED group revealed that women with BED with a comorbid mood disorder reported exposure to a greater number of life events than those without (*χ*_3_^2^ = 11.64, *P* = 0.009). They specifically reported a greater exposure to concerns about safety (50, 30.9% vs. 10, 6.2%; *χ*_1_^2^ = 8.80, *P* = 0.003). There were no other differences on specific life event stressors between BED women with and without comorbid mood disorder (all *P*'s > 0.01).

## 4 Discussion

The results from this study suggest that women with BED experience more antecedent life events in the 12-month period before the onset of disturbed eating than do both psychiatric and non-psychiatric control women of the same age. In addition, the onset of disturbed eating was preceded by particular types of antecedent life events. Increased risk of exposure to life events within the interpersonal domain was closely associated with BED onset. Specifically, significant moves or changes in the family, end of significant relationships or bereavement, experiences of abuse, and critical comments about weight and shape were identified as antecedents of BED onset. In addition, generally elevated experiences of stress at school or work and concerns regarding personal safety suggest that individuals experienced increased vulnerability in the year before BED onset. These results corroborate the theory of social support as a buffer for psychological problems and are consistent with findings that low social support is associated with the onset of binge eating (Stice et al., 1998, 2002) and eating disturbances in female adolescents (for an overview, see Jacobi et al., 2004).

Consistent with risk factor research in eating disorders, most antecedents were of low potency (see Jacobi et al., 2004). However, medium effect sizes were found for exposure to more than three life events, and to the following specific antecedents: 1) critical comments about shape and weight, and 2) work, school or other-related stress. These findings suggest that nonspecific stress coupled with specific criticism related to shape and weight render an individual particularly vulnerable to the development of BED. While exposure to number of life events was related to comorbid depression, exposure to critical comments about weight, shape, or eating was related to obese weight status in the BED group (see moderator analyses). The subjective experience of significant shape and weight criticism is consistent with other findings suggesting that weight and shape concerns play a central role in the development of eating disturbances across the range of eating disorders (Fairburn et al., 1998; Jacobi et al., 2004; Striegel-Moore et al., 2005). In addition, the results of this study provide initial evidence regarding the antecedent life stress that is associated with the onset of BED and other factors that may increase risk of exposure such as depression and obesity.

In terms of specificity of antecedents for BED compared with the psychiatric control group, the only specific factor of medium potency identified was the experience of three or more stressful life events in the year preceding index age. These findings suggest that stressful life events may be associated specifically with the onset of psychological disturbance compared with ongoing psychological disturbances. Although effect sizes were less than medium, certain specific antecedents were identified based on odds ratios (e.g., critical comments about shape and weight and change in family structure). These results add support to the notion that for most individuals the onset of BED is triggered by the accumulation of multiple but relatively low potency variables. Results for BED are similar to the findings of studies of anorexia nervosa (Rastam and Gillberg, 1992) and bulimia nervosa (Welch et al., 1997), which also suggested an accumulation of life events immediately preceding the onset of the eating disorder.

Another notable finding was that antecedent life stressors were varied and to some extent idiosyncratic. When asked whether any other significant experience occurred in the year before the onset of BED, nearly one-third of the women with BED reported something that was not otherwise assessed by the interview. Examples of such stressors included getting married, worsening of parent's drinking, getting engaged, having a sister with an eating disorder that required hospitalization, graduating from college, starting to use drugs, and going to junior high school. These factors are largely social/interpersonal experiences, but they are not necessarily negative events. Nonetheless, they contributed to the experience of feeling overwhelmed, stressed, and diminished in capacity to cope effectively.

This study has significant strengths in that it represents a large, community-based sample of BED, psychiatric and non-psychiatric populations, including an ethnic minority sample. In terms of representativeness of the sample, BMI for the BED group is consistent with previous research documenting the link between BED and obesity. BMI for the NC group fell below representative data for females in the respective age group (Hedley et al., 2004), which, however, is consistent with lower obesity prevalence rates in individuals with higher levels of academic achievement (e.g., Mokdad et al., 2003). This lower rate of obesity may nevertheless be associated with lower levels of exposure to antecedent life stress in the non-psychiatric comparison group (see moderator analysis on obese weight status), thus possibly accentuating greater exposure to life event stress in the BED group.

A further strength of this study consists of the use of semi-structured interviews to ascertain diagnosis and assess antecedents of all cases. For the assessment of antecedents, an index age was carefully established to maximize accuracy in identifying life events that preceded onset of the disorder. However, given the retrospective nature of the assessment, it needs to be noted that precedence could not be absolutely established (Kraemer et al., 1997; Jacobi et al., 2004). Further, the 12-month assessment period was based on the index age of the BED case and was applied to the matched psychiatric case without regard to the onset of the psychiatric disorder of the individual in the psychiatric control group. The rationale for this procedure was to ensure that the same developmental window was assessed across each triplet of BED, psychiatric and non-psychiatric cases; however, its shortcoming is that the relationship between this 12-month window and the onset of the psychiatric case's own disorder remains unclear. Therefore, the comparison between the BED cases and the psychiatric control cases should not be construed as a comparison of antecedents for each disorder but rather must be understood as a comparison of exposure to stressful life events during this 12-month developmental window. A further shortcoming of the psychiatric control group is that it represents a mixture of Axis I diagnoses. In future studies, it would be desirable to assess specific diagnoses (e.g., major depression) with matching on the respective index age to obtain more precise information on antecedents that are associated with a particular disorder. Finally, given the retrospective nature of this case-control design, it would advance the field to study the identified antecedents prospectively.

The findings from this study suggest that in the year preceding onset of disturbed eating, individuals with BED experience an increased number of stressful life events, and these events tend to be interpersonal in nature. Future investigations could explore the psychobiological mechanisms that mediate the relationship between life stress and the onset of BED. Experimental research (Voegele and Florin, 1997) and observational studies (Crowther et al., 2001) have found that stress may trigger binge eating/desire to binge. In these studies, women who binge-eat tended to perceive daily hassles as more stressful than non-binge-eating controls. As results suggest from animal analog studies in rats, stress may magnify restriction-induced overeating for the purpose of stress-reduction as opposed to metabolic purposes (Hagan et al., 2003). However, it is largely unclear whether this applies to humans with BED.

Future investigations that examine and integrate a range of risk and antecedent factors as well as developmental factors have the potential to advance the field (Striegel-Moore, 1993; Wilfley et al., 1997). It is important to consider how these antecedents work together in the onset of BED. It is possible that some mediate or moderate the effect of others. For example, it may be that ending a relationship is associated with major house moves or physical abuse. In addition, future studies examining the moderating influence of coping styles on the impact of potential stressful events could enhance our capacity to predict BED onset (Ghaderi and Scott, 2001; Penley et al., 2002). For example, in a study of anorexic and bulimic symptoms, Troop and Treasure (1997) found that cognitive avoidance was associated with anorexic symptoms whereas cognitive rumination was associated with the onset of bulimic symptoms. Finally, it will be important to examine the relationship between antecedent life stress and personal risk characteristics such as negative self-evaluation and perfectionism.

The findings from this case-control study of antecedents associated with the onset of BED provide direction for future prospective investigations. Continuing this line of research will advance our understanding of the etiology of BED and will empirically inform early intervention or prevention efforts.

## Figures and Tables

**Table 1:** Characteristics for matched samples of women with binge-eating disorder and non-psychiatric control women and of women with binge-eating disorder and psychiatric control women

	BED (*n* = 162)			NC (*n* = 162)					BED (*n* = 107)			PC (*n* = 107)				
	*M*	S.D.	Range	*M*	S.D.	Range	*t*	*P*	*M*	S.D.	Range	*M*	S.D.	Range	*t*	*P*
Index age, years	15.7	7.1	5–35	15.8	7.1	5–35	− 1.50^a^	0.135	14.3	5.5	6–32	14.3	5.5	6–32	− 1.00^d^	0.320
Age, years	30.8	5.8	18–40	30.0	5.6	18–40	3.11^b^	0.002	30.6	5.9	18–40	29.5	6.7	17–49	2.27^d^	0.025
Body mass index, kg/m^2^	34.6	10.0	20.1–69.2	25.6	7.1	16.1–63.5	10.08^c^	< 0.001	33.8	10.0	20.1–68.8	25.9	7.0	17.0–53.4	7.15^e^	< 0.001
				
	*n*	%	*n*	%	*χ*^2^	*P*	*n*	%	*n*	%	*χ*^2^	*P*				
Obesity, BMI ≥ 30.0 kg/m^2^	101	62.7	32	20.0	55.71^f^	< 0.001	61	57.5	23	21.5	25.35^f^	< 0.001				
Education																
High school or less	31	19.1	29	17.9			21	19.6	24	22.4						
Some college	80	49.4	78	48.1			49	45.8	47	43.9						
College graduate or beyond	51	31.5	55	34.0	1.76^g^	0.415	37	34.6	36	33.6	1.02^g^	0.601				

BED, binge-eating disorder; NC, non-psychiatric control group; PC, psychiatric control group; BMI, body mass index. Paired *t* tests; ^a^*df* = 161; ^b^*df* = 159; ^c^*df* = 1, 158; ^d^*df* = 106; ^e^*df* = 105; ^f^McNemar tests, *df* = 1; ^g^Stuart–Maxwell *χ*^2^ tests, *df* = 2.

**Table 2:** Life events occurring within the year before onset of disordered eating in women with binge-eating disorder and within the equivalent year in non-psychiatric or general psychiatric control women, respectively

	BED (*N* = 162)		NC (*N* = 162)		*χ*^2a^					BED (*N* = 107)		PC (*N* = 107)		*χ*^2a^				
	*n*	%	*n*	%	(*df* = 1)	*P*	OR	95% CI	AUC%	*n*	%	*n*	%	(*df* = 1)	*P*	OR	95% CI	AUC%
Major house move	48	29.8	17	10.6	18.37	< 0.001	3.4	1.9–6.2	59.6	30	28.6	19	18.1	2.86	0.091	1.8	0.9–3.5	55.3
Significant episode of physical illness	20	12.3	10	6.2	2.70	0.100	2.1	1.0–4.7	53.1	11	10.4	14	13.2	^b^	0.648	0.8	0.3–1.7	48.6
Pregnancy	16	9.9	10	6.2	^b^	0.286	1.7	0.7–3.8	51.9	4	3.8	5	4.7	^b^	1.000	0.8	0.2–3.1	49.6
Bereavement (close relative/friend/partner)	31	19.1	9	5.6	12.25	< 0.001	4.0	1.8–8.8	56.8	21	19.6	10	9.3	3.70	0.054	2.4	1.1–5.3	55.2
Major episode of illness in close relative/friend/partner	29	17.9	25	15.4	0.21	0.643	1.2	0.7–2.1	51.3	18	17.0	18	17.0	0.00	1.000	1.0	0.5–2.0	50.0
Change in family structure (member leaving or joining)	46	28.6	18	11.2	12.57	< 0.001	3.2	1.8–5.8	58.7	34	32.4	15	14.5	7.90	0.005	3.0	1.5–5.9	59.0
End of relationship with boyfriend/partner	31	19.1	14	8.6	8.26	0.004	2.5	1.3–4.9	55.3	22	20.8	9	8.5	^b^	0.015	2.8	1.2–6.4	56.2
Sexual abuse	14	8.6	5	3.1	^b^	0.064	3.0	1.0–8.5	52.8	10	9.3	10	9.3	^b^	1.000	1.0	0.4–2.5	50.0
Physical abuse	27	16.7	5	3.1	14.70	< 0.001	6.3	2.4–16.8	56.8	19	17.8	17	15.9	0.03	0.855	1.1	0.6–2.3	51.0
Major stress from school, work or other source^c^	91	57.2	39	24.5	31.72	< 0.001	4.1	2.5–6.6	66.4	60	57.6	44	42.3	4.02	0.045	2.0	1.1–3.4	57.7
Critical comments about weight, shape, or eating	69	42.9	14	8.7	38.88	< 0.001	7.4	4.0–13.6	67.1	44	41.5	19	17.9	10.87	0.001	3.3	1.8–6.2	61.8
Safety concerns^d^	60	37.0	18	11.1	24.72	< 0.001	4.7	2.6–8.4	63.0	40	37.7	22	20.8	5.78	0.016	2.4	1.3–4.4	58.5
Anything else significant	50	31.4	18	11.3	17.16	< 0.001	3.7	5.0–6.6	60.1	33	31.7	15	14.4	7.23	0.007	2.9	1.4–5.7	58.7

BED indicates binge-eating disorder; NC, non-psychiatric control group; PC, general psychiatric control group; OR, odds ratio; 95% CI, odds ratio's 95% confidence interval; AUC%, area under the curve percent. ^a^McNemar tests; ^b^exact test; ^c^collapsed from two items: 1) major pressure or stress at school or work; 2) any other stress or pressure; ^d^collapsed from four items: 1) worried for personal safety; 2) feeling unsafe at home; 3) feeling unsafe in your neighborhood, 4) feeling unsafe at school or work.

**Table 3:** Number of life events occurring within the year before onset of disordered eating in women with binge-eating disorder and within the equivalent year in non-psychiatric or general psychiatric control women, respectively

	BED (*n* = 162)		NC (*n* = 162)		Post hoc^b^	*P*	OR	95% CI	AUC%	BED (*n* = 107)		PC (*n* = 107)		Post hoc^b^	*P*	OR	95% CI	AUC%
	*χ*_3_^2^^a^ = 64.19, *P* < 0.001									*χ*_3_^2^^a^ = 22.28, *P* < 0.001								
	*n*	%	*n*	%						*n*	%	*n*	%					
No events	10	6.2	65	40.1	41.44	< 0.001	0.1	0.0–0.2	33.1	8	7.5	19	17.8	5.26	0.022	0.4	0.1–0.8	44.9
One event	20	12.3	38	23.5	7.71	0.006	0.5	0.3–0.8	44.4	10	9.3	26	24.3	48.53	0.004	0.3	0.1–0.7	42.5
Two events	34	21.0	25	15.4	1.98	0.160	1.5	0.8–2.6	52.8	22	20.6	29	27.1	1.20	0.274	0.7	0.4–1.3	46.8
Three or more events	98	60.5	34	21.0	49.95	< 0.001	5.8	3.5–9.4	69.8	67	62.6	33	30.8	19.93	< 0.001	3.9	2.2–6.9	65.9

BED indicates binge-eating disorder; NC, non-psychiatric control group; PC, general psychiatric control group; OR, odds ratio; 95% CI, odds ratio's 95% confidence interval; AUC%, area under the curve percent.

a Stuart-Maxwell *χ*^2^ test.

b McNemar tests (*df* = 1).
